# 174. Evaluation of FilmArray® Blood Culture Identification and TheraDoc® Utilization at a Veterans Affairs Health Care System

**DOI:** 10.1093/ofid/ofac492.252

**Published:** 2022-12-15

**Authors:** Galina Wang, Bailey Guest, Madison Treadway, David Rudd, Kelly Sugarman

**Affiliations:** Clement J. Zablocki VA Medical Center, Milwaukee, Wisconsin; Salisbury VA Health Care System, Salisbury, North Carolina; Campbell University, Buies Creek, North Carolina; Salisbury VA Health Care System, Salisbury, North Carolina; Salisbury VA Health Care System, Salisbury, North Carolina

## Abstract

**Background:**

Bacteremia is associated with significant morbidity and mortality, with each hour of delay initiating antibiotics associated with a 7.6% decrease in survival rate. At the Salisbury Veterans Affairs Health Care System (VA HCS), FilmArray® Blood Culture Identification (BCID) is used along with TheraDoc® to assist with selection of antimicrobial regimens as an antimicrobial stewardship practice at the site. The purpose of this study is to evaluate the time to appropriate antibiotic therapy based on positive BCID results.

**Methods:**

This study is a retrospective, quality assurance project conducted at a 284-bed VA medical center from May 2018 through December 2020 of Veterans with positive BCID results identified on TheraDoc®. The primary endpoint is to identify the mean time for initiation of appropriate antimicrobial therapy defined as at least 80% susceptibility on local antibiogram to the resulting BCID organism for Veterans not on antibiotics or on inappropriate therapy from the time of BCID positivity. Secondary endpoints include comparing time to appropriate therapy with or without Infectious Diseases (ID) and Antimicrobial Stewardship Program (ASP) coverage, time to de-escalation, and contributors to time delays.

**Results:**

A total of 168 Veterans were included in the study with 138 Veterans (82%) receiving appropriate antibiotic therapy at the time of BCID results and 30 Veterans (18%) on inappropriate or no empiric antibiotics. The mean time to appropriate therapy of the 30 Veterans on inappropriate or no antibiotics at the time of BCID positivity was 13 hours and 54 minutes with provider order entry being the largest factor in time delays. Of these 30 Veterans, BCID positivity occurred in 6 Veterans during business hours with onsite availability to ID and ASP to which the mean time to appropriate therapy was 3 hours and 29 minutes compared to 16 hours and 30 minutes for the 24 Veterans with results outside of business hours. For the 138 Veterans on appropriate therapy, the average time to de-escalation was about 43 hours and 52 minutes.

Percentage of ID and/or ASP involvement.

**Figure:**
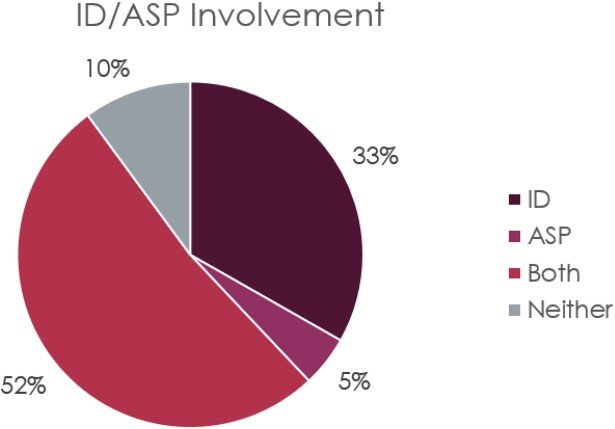

This pie chart shows the percentages of Veterans with ID and/or ASP involvement.

Time to de-escalation.

This graph demonstrates the time to de-escalation for Veterans on appropriate therapy at the time of BCID positivity.

Inappropriate Therapy Group.

**Figure:**
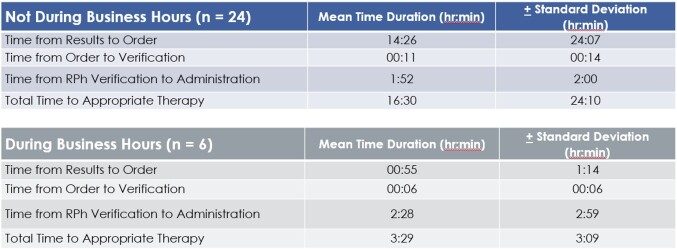

This graph compares time to appropriate therapy between results occurring during business hours or outside of business hours for Veterans on no initial antibiotics or inappropriate therapy.

**Conclusion:**

BCID and TheraDoc® are helpful antimicrobial stewardship tools to assist with reducing time to appropriate therapy when consistently monitored. Additional resources and education may be of benefit to providers covering outside of business hours.

Example of BCID Algorithm

**Figure:**
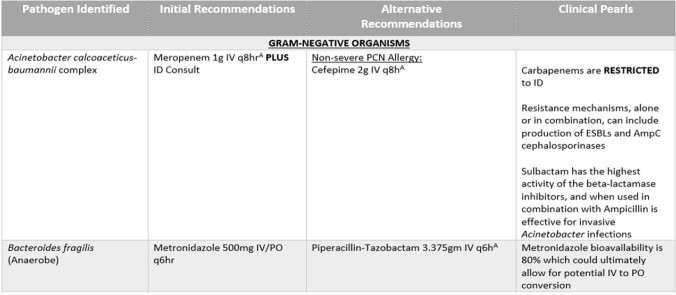

Creation of a local BCID algorithm has been started as a result of this study. The goal of this resource is to be a tool for providers to help guide therapeutic decisions when ID and/or ASP are not readily available.

**Disclosures:**

**All Authors**: No reported disclosures.

